# Commentary: Grounded procedures: A proximate mechanism for the psychology of cleansing and other physical actions

**DOI:** 10.3389/fpsyg.2020.02137

**Published:** 2020-09-02

**Authors:** Tobias Otterbring, Panagiotis Mitkidis, Lene Aarøe, Christian T. Elbæk

**Affiliations:** ^1^Department of Management/MAPP, Aarhus University, Aarhus, Denmark; ^2^Department of Management, Aarhus University, Aarhus, Denmark; ^3^Center for Advanced Hindsight, Duke University, Durham, NC, United States; ^4^Department of Political Science, Aarhus University, Aarhus, Denmark

**Keywords:** domain specificity, morality, evolutionary psychology, cleansing effects, disgust

Lee and Schwarz (2020) present five falsifiable predictions derived from their grounded procedures account and state that if grounded procedures serve as a proximate mechanism for cleansing effects, then cleansing should decrease or erase the otherwise observed impact of a prior event (1) across domains and (2) across valences. Furthermore, they postulate that (3) cleansing manipulations that more strongly engage sensorimotor capacities should have a particularly powerful influence, that (4) psychological antecedents of cleansing should be valence-asymmetric, such that motivation for cleansing as a procedure for separation should be triggered more easily by negative (vs. positive) valence, and, finally, that (5) conceptually similar effects should extend from cleansing to other forms of separation and connection. While we perceive each of these premises as plausible, we wanted to focus our commentary not so much on what the authors do state, but rather on one aspect that they do not specify, whose elaboration would further facilitate falsifiability.

Specifically, we would have liked the authors to clearly communicate whether they assume domain-specific cleansing effects to be stronger than effects in unrelated or only symbolically similar domains. For instance, some but admittedly not all acts of separation are likely induced through an aversive state (e.g., immoral behaviors being erased through cleansing in order to “wash away the sins” and reduce the saliency of an aversive state of arousal). Other aversive states, such as acute hunger, have shown to exert stronger effects on domain-specific responses, while still having some, albeit weaker effects in other domains (for a meta-analysis, see Orquin and Kurzban, 2016). For example, hungry (vs. satiated) individuals are particularly prone to favor hedonic (vs. utilitarian) food options, but also exhibit a similar, but weaker tendency to prefer other hedonic options that have nothing to do with food (Otterbring, 2019). Based on such findings, we suspect that cleansing effects will (1) have the strongest impact in domain-specific situations, while the strength of these effects should (2) attenuate in domains that are only symbolically similar (i.e., conceptually related but not domain specific, such as certain religious rituals meant to create a pure conscience; Xygalatas et al., 2013; Mitkidis et al., 2017), and (3) further decrease in domains that are entirely unrelated to disgust, morality, purity, divinity, virginity, and other conceptually connected phenomena. In our view, these assumptions would align with a deep-rooted, ultimate (as opposed to proximate) account, as such a strength ranking of responses, ranging from strongest in domain-specific situations, through weaker in symbolically (and conceptually) similar domains, to weakest in unrelated domains appears adaptive and, consequently, something that may have evolved throughout human history (Cosmides and Tooby, 1994; Duchaine et al., 2001; Kirkpatrick et al., 2002; Kanazawa, 2004). Thus, while the authors delineate their expected strengths of cleansing effects as a function of whether they relate to the self (vs. other) as the agent and whether the self (vs. other) is the patient, we wonder if and why they do or do not predict differentially strong cleansing effects as a function of domain specificity.

## Author Contributions

TO lead-authored the article, with input from PM, LA, and CE. All authors approved the final version of the article prior to submission.

## Conflict of Interest

The authors declare that the research was conducted in the absence of any commercial or financial relationships that could be construed as a potential conflict of interest.
